# TVB-EduPack—An Interactive Learning and Scripting Platform for The Virtual Brain

**DOI:** 10.3389/fninf.2015.00027

**Published:** 2015-11-25

**Authors:** Henrik Matzke, Michael Schirner, Daniel Vollbrecht, Simon Rothmeier, Adalberto Llarena, Raúl Rojas, Paul Triebkorn, Lia Domide, Jochen Mersmann, Ana Solodkin, Viktor K. Jirsa, Anthony Randal McIntosh, Petra Ritter

**Affiliations:** ^1^Minerva Research Group BrainModes, Max Planck Institute for Human Cognitive and Brain SciencesLeipzig, Germany; ^2^Department of Neurology, Charité – University MedicineBerlin, Germany; ^3^Bernstein Focus State Dependencies of Learning and Bernstein Center for Computational NeuroscienceBerlin, Germany; ^4^Intelligent Systems and Robotics Lab, Department of Mathematics and Computer Science, Free UniversityBerlin, Germany; ^5^CodemartCluj-Napoca, Romania; ^6^CodeBox GmbHStuttgart, Germany; ^7^Departments of Anatomy & Neurobiology and Neurology, School of Medicine, University of California, IrvineIrvine, CA, USA; ^8^Institut National de la Santé et de la Recherche Médicale, Institut de Neurosciences des Systèmes UMR 1106, Université d'Aix-MarseilleMarseille, France; ^9^Rotman Research Institute of Baycrest Centre, University of TorontoToronto, ON, Canada; ^10^Berlin School of Mind and Brain and Mind and Brain Institute, Humboldt UniversityBerlin, Germany

**Keywords:** The Virtual Brain, brain modeling, computational neuroscience, connectome, educational platform

## Abstract

The Virtual Brain (TVB; thevirtualbrain.org) is a neuroinformatics platform for full brain network simulation based on individual anatomical connectivity data. The framework addresses clinical and neuroscientific questions by simulating multi-scale neural dynamics that range from local population activity to large-scale brain function and related macroscopic signals like electroencephalography and functional magnetic resonance imaging. TVB is equipped with a graphical and a command-line interface to create models that capture the characteristic biological variability to predict the brain activity of individual subjects. To enable researchers from various backgrounds a quick start into TVB and brain network modeling in general, we developed an educational module: TVB-EduPack. EduPack offers two educational functionalities that seamlessly integrate into TVB's graphical user interface (GUI): (i) interactive tutorials introduce GUI elements, guide through the basic mechanics of software usage and develop complex use-case scenarios; animations, videos and textual descriptions transport essential principles of computational neuroscience and brain modeling; (ii) an automatic script generator records model parameters and produces input files for TVB's Python programming interface; thereby, simulation configurations can be exported as scripts that allow flexible customization of the modeling process and self-defined batch- and post-processing applications while benefitting from the full power of the Python language and its toolboxes. This article covers the implementation of TVB-EduPack and its integration into TVB architecture. Like TVB, EduPack is an open source community project that lives from the participation and contribution of its users. TVB-EduPack can be obtained as part of TVB from thevirtualbrain.org.

## Introduction

The Virtual Brain (TVB; thevirtualbrain.org) is a Python-based neuroinformatics platform for the simulation of realistic large-scale networks (Jirsa et al., 2010) of neural population models available for all major operating systems, including Windows, Mac OS, and Linux (Ritter et al., 2013; Sanz-Leon et al., 2013; Roy et al., 2014; Woodman et al., 2014). TVB provides a generic framework for computational modeling and tools to extract structural and functional connectomes from multimodal datasets (Schirner et al., 2015) in order to link personalized brain network structure with neural population models. It is an open-source whole-brain simulation platform that integrates empirical data of different modalities and spatial scales to construct comprehensive and biologically plausible models of global brain network dynamics. TVB combines structural magnetic resonance imaging (MRI) data (to account for brain geometry) with diffusion-weighted MRI (to account for brain connectivity) to establish an individualized brain network model. Brain connectivity as defined in the context of brain network models (Sanz-Leon et al., 2015) comprises the complete space-time structure of the large-scale connectome, i.e., time delays and connection strengths between brain regions. Computational models in TVB generate functional data that can be directly compared against empirical data such as functional MRI, electroencephalography (EEG), magnetoencephalography (MEG), intra-cortical local field potentials (LFP), and multiunit activity (MUA) recordings (Ritter et al., 2013; Roy et al., 2014). These empirical data serve as targets to modify the free biologically meaningful parameters of the model in order to produce optimal model fit. The parameters are either constrained by biology, or by dynamics considerations. The neuroinformatics architecture of TVB is used to create large sets of brain models within a unifying framework, to systematically explore the relation between structural network features and biophysical parameters to different empirical brain states or network patterns. TVB relates the signals of different imaging modalities to underlying neuronal sources and thereby provides insight into underlying mechanisms (Becker et al., 2015). While currently TVB accounts for brain structure and function, it will next also accommodate detailed anatomical maps, e.g., of receptor distributions as additional model constrain adding another level of detail and bringing the model's behavior closer to the real brain. Like all models, brain network models use abstractions to describe neuronal population behavior and their interaction. Despite the reproduction of several features of empirical neuroimaging data, their biological plausibility is still a matter of ongoing research.

Obtained as standalone software, TVB is distributed including all used dependencies, such as the Python interpreter, CherryPy, Genshi, NumPy, and SciPy. Optionally controlled by a convenient graphical user interface (GUI) or a Python programming interface for advanced modeling and post-processing, TVB appeals to a wide user group. Running in a web browser, 2D and 3D visualizations are implemented with WebGL and scalable vector graphics; data and project management is handled by an SQL database. As a community-driven open-source software, TVB is licensed under GPL 2.0 and sources may be freely forked from a GitHub repository, modified, and distributed.

In TVB, brain structure is parcellated into cortical and subcortical regions with each region serving as a node in a large-scale network model. Edges of the network represent the anatomical connectivity between regions, mediated by long-range white matter fiber bundles. The local activity at each node is simulated by models that capture typical dynamics of mesoscopic neuronal population activity, involving interacting excitatory, and inhibitory subpopulations. Regions are connected by edges that represent the strengths and time-delays of interactions between anatomical regions, which are inferred from diffusion-weighted magnetic resonance imaging (*dw*-MRI) data by tractography. Based on subject-specific anatomy, TVB enables the full-brain simulation of individualized mean field, EEG, MEG, and functional MRI (fMRI) activity. Local node models comprise a choice of more than 10 population models that, depending on their parameterization, enable the simulation of brain activity under different physiological and pathological conditions.

Despite a set of visual aids that make TVB understandable for the user, it is still a complex simulation tool. Specially, when considering users without deep knowledge in neuroscience and its computational principles. Therefore, in order to facilitate TVB's usability of the Graphical User Interface (GUI), an educational tool that could help the user to learn interactively was required. Here we introduce TVB-EduPack (Matzke, 2014), a new module of TVB that serves a two-fold purpose:

- First, to enable a quick start into the variety of model construction, simulation, post-processing, and analysis options available, TVB-EduPack offers an interactive guide that leads users through functionality, handling, and application of TVB. Interactive tutorials guide the user in a step-by-step manner through use case examples that show how to perform advanced modeling with TVB. Using this instructional design framework, TVB-EduPack combines didactic preparations of computational neuroscience contents with practical results delivered by brain simulation.
- Second, in order to allow the TVB users to automate or demonstrate their work, TVB-EduPack contains a graphical script creation tool that exports simulation and model configurations and thereby enables users to create re-usable and modifiable batch scripts for the TVB console interface.

EduPack tutorials are divided into two groups addressing new and experienced users. Introductory tutorials familiarize new users with basic TVB usage, while advanced tutorials offer guidance in use-case scenarios. Another application is the convenient formalization and reproduction of published results enhancing reproducibility and visibility. The set of TVB-EduCases is constantly growing with new EduCases being added in an ongoing manner, as they are flexible and convenient to create using XML Schema Definitions. EduCases can be easily generated on the basis of TVB project files. EduPack exports the individual steps of a regular TVB application like model construction and simulation as XML file that can be enhanced by guiding comments, videos, images or audio files. EduCases encompass a variety of user backgrounds, interests and skill sets. Tutorials and educational elements can be configured to lay the focus on, e.g., physiological, pathological, neuroscientific, or theoretical contents.

While GUIs are convenient to use, their possibilities and flexibility are generally limited. As modeling scenarios get more complex, users often switch to the programming interface. To ease this transition, EduPack contains a script generation tool that automatically exports simulation script templates with model parameter and simulator configuration settings specified within the GUI.

In addition to the newly developed educational framework, TVB distributions contain a user guide and an online documentation for the GUI and the programming interface, which also includes some tutorials and information about data structures. Parts of the user guide are also embedded into the GUI in the form of clickable help buttons that reveal contextual information in the form of GUI overlays. Furthermore, TVB hosts a mailing list as support and discussion forum and to get into direct contact with the developers. While earlier articles on TVB focused on the description of the mathematical framework, the software infrastructure and analyses of dynamics of brain network models, here we now introduce an educational add-on module for TVB that enables new users to quickly familiarize themselves with TVB usage and brain network modeling in general.

## Functionality and design

### Background: Modeling brain activity with TVB simulation platform

TVB-EduPack is an educational module for the software product TVB. TVB is a neuroinformatics platform that enables the GUI or command based construction, simulation and analysis of brain network models (BNMs). BNMs are mathematically represented by a generic large-scale brain network equation (Jirsa, 2009; Spiegler and Jirsa, 2013) that describes how each node of the large-scale network is governed by its own intrinsic dynamics and the interaction with all connected nodes. TVB distinguishes two types of interactions: instantaneous local interactions whose spatial extent is described by a Kernel function and long-range interactions. While in the real system long-range interactions are mediated by groups of axons that are bundled to form white-matter fibers, in TVB, global node-node communication is formalized by two connectivity matrices: a weight matrix that specifies the strength of information transmission and a delay matrix that specifies the time delays due to signal transmission delays. The activity of nodes is described by neural mass models that approximate intrinsic population dynamics, e.g., Wilson-Cowan (Wilson and Cowan, 1972), reduced representations of excitatory and inhibitory networks of Fitz-Hugh Nagumo (FitzHugh, 1961), Hindmarsh-Rose (Stefanescu and Jirsa, 2008), and Wong-Wang oscillators (Wong and Wang, 2006; Deco et al., 2009), Jansen-Rit (Jansen and Rit, 1995), Kuramoto (Kuramoto, 1975; Cabral et al., 2011), and a generalized 2d oscillator capable of generating a wide range of population dynamics on multiple time-scales including multistability and coexisting oscillatory and non-oscillatory regimes.

**Figure 1:**
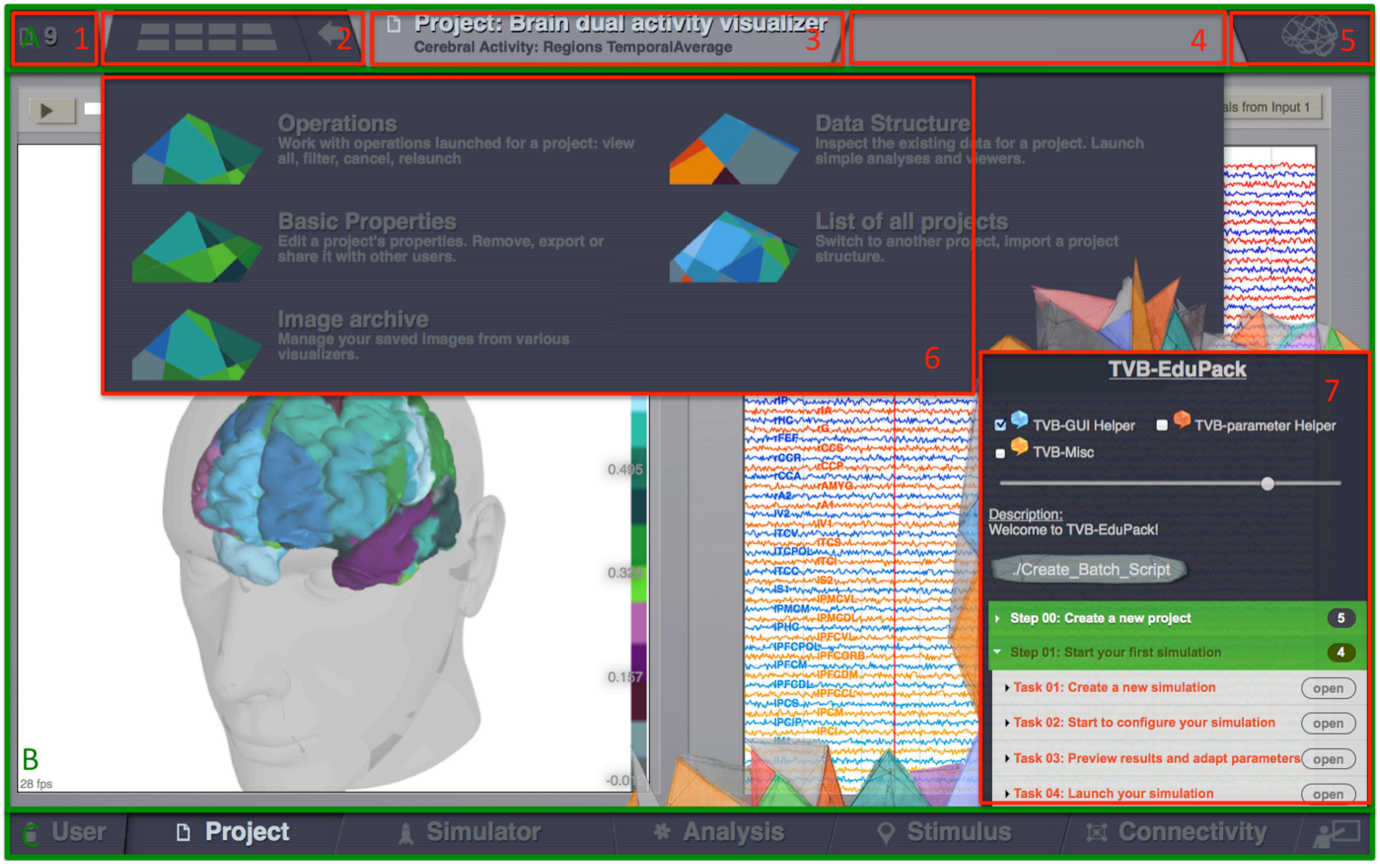
**The Virtual Brain GUI is divided into three main areas: header menu (A), main area (B), and footer menu (C)**. The header menu shows the identifier of the current project (1), a button (2) a drop down menu (6) with functionalities of the active tab (3). The upper right corner contains an information panel about currently running operations (4) and another drop-down menu (5) with options for the current tab. The main area shows the currently active TVB interface-tab and, if active, the Edu-Pack menu (7). The footer **(C)** allows to switch between the different interface tabs: **USER**, for managing User accounts and general settings, **PROJECT**, for managing and exchanging projects and associated input/output data, **SIMULATOR**, to configure, launch, and monitor simulations, **ANALYZE**, where simulation results can be analyzed, **STIMULUS**, where users can create model stimulation patterns for simulations and **CONNECTIVITY**, for visually editing local and large-scale connectivity.

TVB GUI is divided into three main areas (Figure 1): Header menu (Figure 1A), main area (Figure 1B) and footer menu (Figure 1C). Starting at the top left corner, the header menu shows the identifier of the current project (1). A button (2) opens a drop down menu (6) for the currently running GUI interface. The drop down menu contains links to functionalities implemented in different interfaces as described in the TVB user guide and online documentation. The subsequent panel shows status information about current operations (3 and 4) followed by another drop-down menu (5) that allows setting options for the current interface. The footer (Figure 1C) switches between the different interfaces of TVB: **USER**, for managing user accounts and general settings, **PROJECT**, for managing and exchanging projects and associated input/output data, **SIMULATOR**, to configure, launch and monitor simulations, **ANALYZE**, where simulation results can be analyzed, **STIMULUS**, where users can create model stimulation patterns for simulations and **CONNECTIVITY**, for visually editing local and large-scale connectivity.

Both, GUI and programming interface act as front-ends for TVB. Following a client-server model, both interfaces connect to the **Flow Manager** that controls those back-end modules and storage components (the GUI via a web-server), enabling multi-user applications on PC or supercomputers. The **Web Server** generates the HTML interface by receiving data from and passing inputs to the flow-manager. The **Flow Manager** is in charge of TVB's high-level program control flow and regulates the interaction of all other back-end modules by receiving user actions, feeding back of TVB's responses and all internal processing. The **Database**, alternatively SQLite or PostgreSQL, organizes project structures by storing references to involved data entities like time series, connectivity matrices, surface triangulations or sensor coordinates. Actual data are stored as HDF5 or ASCII files, due to size. The storage component of TVB is optional and can be switched off when using the console interface.

To implement TVB's complex graphical interface, the **HTML Interface** consists of several client-side components: **Data Reader**, feeds data from the back-end to the front-end; mainly needed for asynchronous calls toward the server; **WebGL Renderer**, for displaying 3d objects in canvas, **Graph Renderer**, for displaying 2d plots, mainly with SVG. All TVB pages in the web GUI are generated based on Genshi templates on the server side and manipulated on the client-side by JavaScript. The important elements of the generated pages have unique identifiers that are used by TVB-EduPack to retrieve position, status and content of GUI elements and to control or interact with the desired objects in the DOM-tree (Document Object Model tree; a structure for organizing objects in HTML documents) of the respective page.

### TVB-EduPack functionality

#### Requirements

The main idea of TVB-EduPack is to provide new users with a tool that enables them to quickly and easily familiarize themselves with TVB and to use it effectively. Experienced users learn to use the more advanced and sometimes not-so-obvious possibilities of working with TVB and to create re-usable and modifiable batch scripts for the command interface. It also serves as an instrument to quickly assess the dynamics of typical brain network activity as it shows up on different scales (e.g., population level, network level), in different imaging modalities (e.g., LFP, EEG, fMRI, MEG, stereotactic EEG) and using different commonly used metrics (e.g., Functional Connectivity, Power Spectral Density, Effective Connectivity). Prior to implementation, several requirements regarding functionality have been specified for TVB-EduPack:

- Comprehensible interface that seamlessly integrates into TVB GUI
- Simultaneous execution of TVB and TVB-EduPack allowing users to start EduPack programs at all times during TVB usage
- Walkthrough tutorials that guide users through the interface by pinpointing the sequence of required steps for different tasks within TVB's GUI
- Notification of futile simulation configurations
- Tutorials that show increasingly complex usage scenarios
- Knowledge transfer, mentorship and computational neuroscience training by providing interactive guided experiences that impart dynamical systems behavior
- Convenient authoring of new EduPack tutorials
- Facility for creating reusable TVB command interface scripts based on simulations or analyses that have been created with the GUI
- Customized support and educational contents for different user groups

Aforementioned requirements have been implemented as two core functionalities of TVB-EduPack, explained in the following.

#### Interactive guides and tutorials

**Figure 2:**
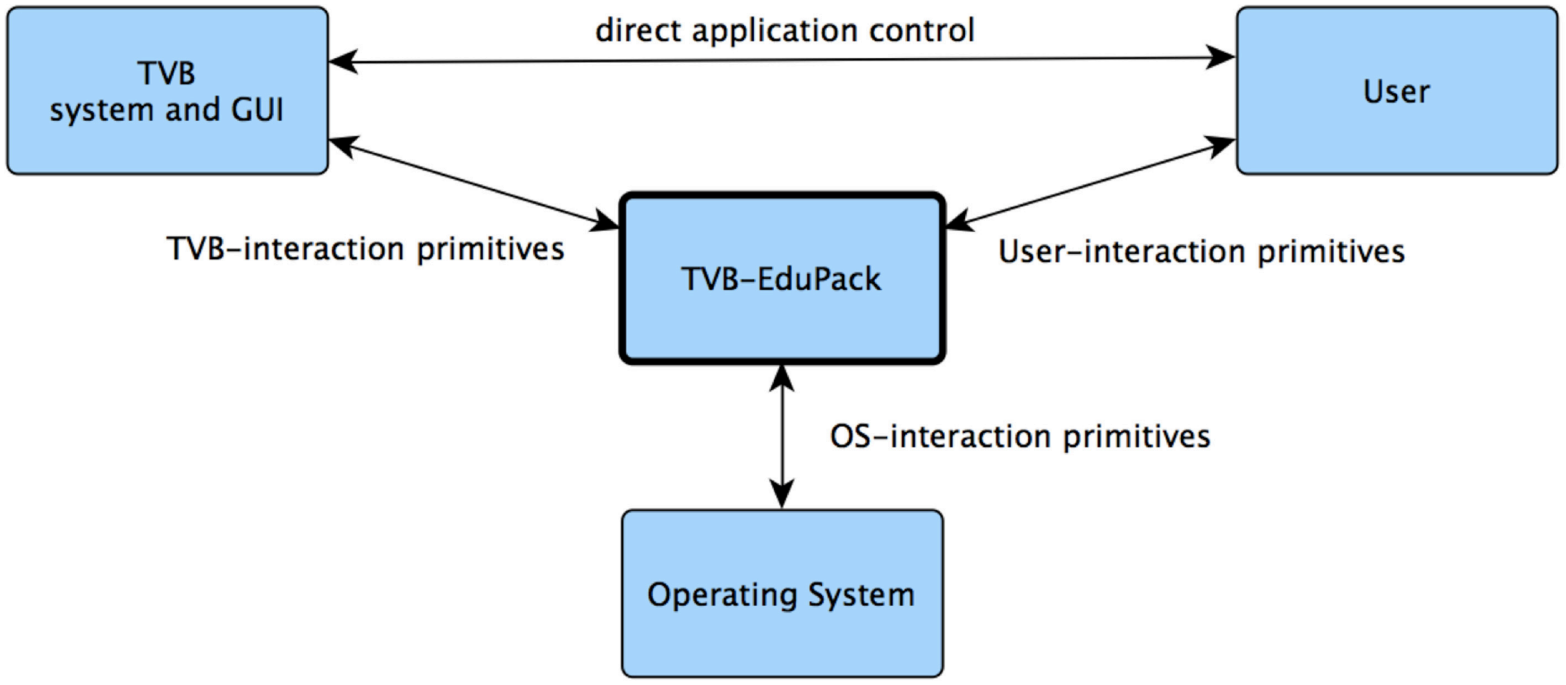
**EduPack's two-way interaction architecture**.

The complexity of the human brain is reflected within the models and tools used for simulating it. To enable a quick start into working with TVB and BNM in general, EduPack includes interactive tutorials that guide through functionality of the software and related computational neuroscience topics. In contrast to the documentation and reference, EduPack tutorials allow users to directly engage in complex modeling scenarios while learning to use TVB. One fundamental requirement was to automate the operation of TVB while letting the user the possibility to maintain full control over TVB (Figure 2). Therefore, at any arbitrary point every EduPack-tutorial can be paused or completely stopped allowing the user to continue with alternative actions. EduPack tutorials can be used as a reference for complicated use-case scenarios, since users are also not obligated to follow the linear structure of tutorials, but can also skip steps and pick only actions that are relevant for the intended usage (except when preconditions for those actions need to be fulfilled). At relevant positions, topics that concern the mechanics of BNMs and computational neuroscience in general are explained with the help of short animations, videos, or references to further material. Different types of graphical elements, dubbed Helper Elements and Action Primitives, add links to additional information at GUI positions, emphasize the sequence of actions or highlights the elements that are relevant for a certain task; furthermore, they verify the actions of the user and give interactive feedback in the case of incorrect usage, e.g., when meaningless parameter settings have been used or relevant information is missing in the context of the current EduCase scenario. For example, the software informs users when parameters of a model are set such that they will not reproduce the desired results of the active tutorial. Generic XML structures allow for flexible modification and extension of these elements, enabling the community to create new tutorials or enhance the reproducibility and visibility of results.

The educational component of TVB-EduPack realizes two different educational purposes:

- *EduStart*, which gives users an interactive introduction into the software TVB while introducing concepts and methodologies in computational neuroscience.
- *EduCase*, which develops computational neuroscience concepts and methods further by leading the user through in-depth tutorials that exemplify typical applications like exploring dynamical regimes of different models or tuning parameters to reproduce specific types of neuronal activity.

TVB-EduStart tutorials teach users how to work with TVB. Users are first introduced into the basic functionality and usability, like creating projects, importing subject specific data and constructing brain network models. At pivotal positions of the GUI corresponding tutorials can be started upon pressing the button. While guiding users through the different steps of different software functions, EduPack provides the users background information about the steps they perform and introduces relevant concepts of computational neurosciences in a step-by-step manner. TVB-EduCase tutorials lead users through the wide spectrum of functionalities up to the point of being able to generate and simulate models and to post-process and analyze data provided by TVB.

A TVB-EduCase refers to a specific use case in TVB; therefore, it is composed of the following elements:

- one or more **steps** and **sub-steps** which describe and guide the use case
- one or more **preconditions** for a use case, step or sub-step
- one or more **tasks** that need to be fulfilled within a step or sub-step before being able to continue to the next one
- one or more assigned **actions** that need to be performed within every step or sub-step

Thereby, this structure, implemented as XML file, enables the creation of interactive tutorials and automatically running macros. This automated application control uses the same set of actions that are also used for the tutorials, so that tutorials and macros can be interchanged flexibly.

#### Command interface script creator

TVB-EduPack provides a user-friendly way to create scripts for the command interface of TVB based on simulations that were set up within the GUI. This feature enables users to automatically export scripts that can be used as input for the console interface of TVB, after model and simulation settings have been configured within the GUI. Thereby, the script generator allows users to quickly learn the structure and usage of the command line interface. While the GUI is easier to use for beginners, the console interface allows more flexibility, since actions are not confined by the operations implemented in the GUI. Script files have the advantage that they can be quickly modified and customized as required; all specified data sets, parameters, and model configurations can be exchanged by text replacement, allowing rapid batch processing. For example, after creating a specific simulation scaffold and exporting it as a script, the user can define a large number of structural connectivity datasets for which the operations are to be performed. Regions for parameter sweeps can be easily adapted and local models flexibly exchanged. Since the interface is specified in Python, users can take advantage of the full power of a high-level programming language and its huge collection of data analysis toolboxes for post-processing, data management and analysis.

### Interface design

The major design requirements and constraints for TVB-EduPack are: (i) smooth integration into the existing TVB GUI and the TVB framework in accordance with its own design principles, (ii) neglectable interference with the performance, functionality, and usability of TVB, (iii) easy maintainability and flexibility regarding extension, (iv) streamlined realization of intended functionality, (v) easy and pleasant usage. However, in order to incorporate all functionalities described in the previous section, it was necessary to reach a compromise between a user-friendly design that interferes with the main parts of TVB GUI as little as possible, while at the same time being able to cover the full range of desired features. A further constraint was the capability of the interface to be invoked at any time during the regular TVB work process. While it is necessary that EduPack is always available if needed, it should still permit general interaction between TVB and users and not hamper any of the features. Consequently, EduPack was integrated as an overlay, thus, enabling its visibility in all interfaces of TVB (Project, Simulator, etc.). A new button was added to the right end of the footer menu that enables the on-demand activation or deactivation of EduPack at any moment during TVB usage.

**Figure 3:**
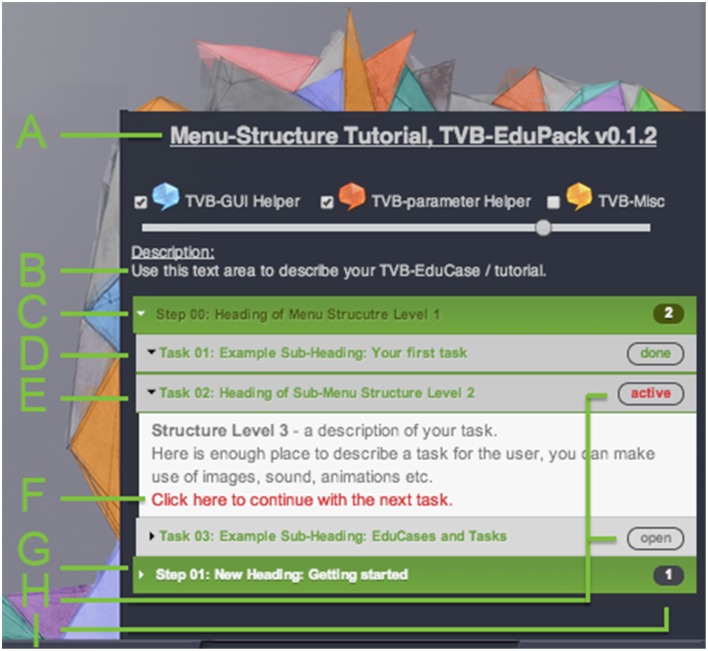
**Lower right section of the TVB Connectivity interface with activated TVB-EduPack in Tutorial Mode and with loaded example tutorial (See main text for label description)**.

Figure 3 shows the design of the tutorial view of EduPack activated within TVB Connectivity interface. EduPack consists of a window overlay with attached and partially transparent hand-drawn images that playfully pick up the TVB logo design to accentuate the contrast between TVB and TVB-EduPack, but also to show its affiliation. This design concept of comic style images and animations is recurring through all TVB-EduPack elements to express the didactic idea and to contrast it with the rigorous scientific style of TVB. The size of the overlay rescales with the browser size and is slightly transparent so that the user still sees the underlying TVB GUI.

**Figure 4:**
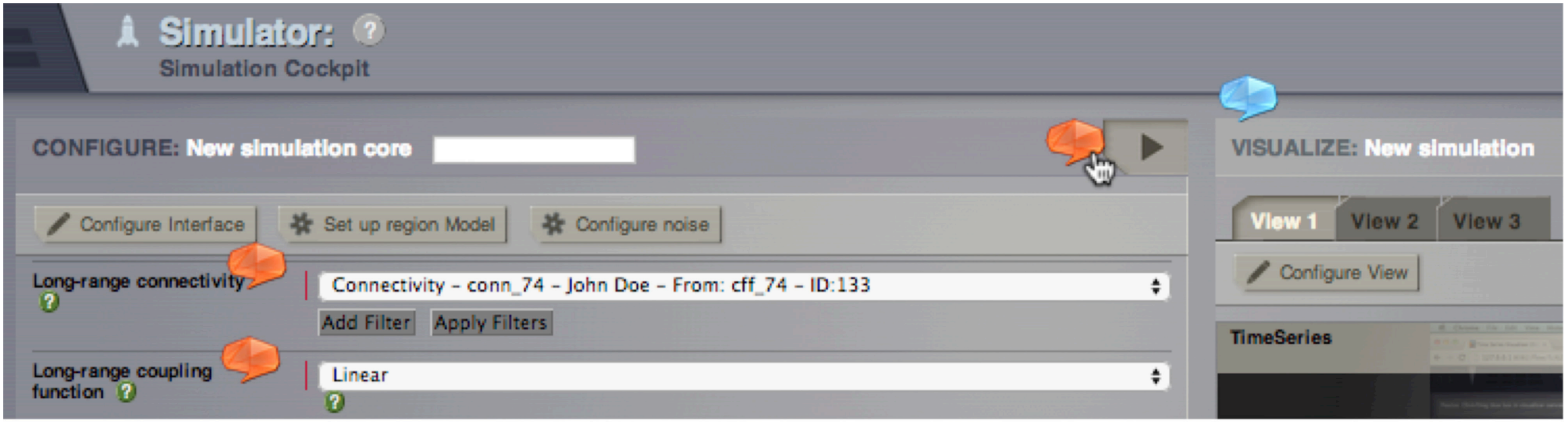
**Section of TVB Simulator interface with integrated parameter Helper Elements (red) and TVB-GUI Helper element (blue)**.

Checkboxes in the upper part activate or deactivate different types of Helper Elements in the TVB GUI. Helper Elements are clickable dialogue balloons that appear next to important elements of TVB GUI (Figure 4) and a slider that controls their transparency. Three types of Helper Elements are included:

1. TVB-GUI Helper (blue): These elements point to TVB GUI elements and provide contextual information about different objects, like buttons, frames, or visualizers.
2. TVB-Parameter Helper (red): The parameter Helper Elements impart physiological interpretations and further background information for the different parameters and models that are available within the TVB Simulation page.
3. TVB-Misc Helper (yellow): The miscellaneous Helper Elements describe all software elements that are not part of the other groups.

The main tutorial menu is located below the Helper element control panel. The steps of a tutorial are indicated by green boxes in the main tutorial menu. Using an accordion design, boxes expand and reveal their content (e.g., tasks, sub-steps or textual descriptions) when clicked.

Since the size of the TVB-EduPack is fixed a scrollbar appears if more vertical space is needed. In case that the overlay window lies on top of relevant TVB elements, EduPack can be shifted to another position by using a so-called action primitive described later.

All design elements of the EduPack interface are defined in CSS groups and are dynamically manipulated by corresponding JavaScript functions at runtime. Tutorials are specified by XML documents that encode a generic procedural protocol and enable proper structure of created tutorials. A generic XML Schema Definition for creating and manipulating tutorials was developed and is described in the following.

A tutorial consists of three structure levels that describe its individual steps and additional meta-information. The first level is called *Step* and groups one or more second-level elements called *Sub-Steps*. *Steps* and *Sub-Steps* can contain instructions for the user, Action Primitives, constraints, or other featured elements.

The following list contains a description of tutorial elements as shown in Figure 3 and their corresponding XML tags.

- Title of tutorial and version of TVB-EduPack (<*title*>).
- Introductory description text for loaded EduCase (<*description*>).
- Structure level 1: A <*step*> element groups one <*step-id*> element (starting at *00*), one title element <*label*>, and one or more <*sub-step*> elements. In the figure the element is active and displays three sub-steps.
- Structure level 2: Completed <*sub-step*> element. A <*sub-step*> element contains one <*substep-id*> element and one or more <*task*> elements. The heading color relates to the type (indicated by <*type*> element) of the sub-step—allowed values are: “description” (green), “parameter” (*red*) and “misc” (*yellow*). The tutorial sub-step was successfully completed, indicated by the text “done.”
- Structure level 2: Active <*sub-step*> element.
- Structure level 3: <detail> elements contain sub-step description text. It can contain HTML elements like images or integrated video components.
- A <*sub-step*> element can have an auto-finish constraint that performs the described action of the sub-step, or as shown here, contain a link that will proceed to the next element and mark the element as finished after clicking it.
- Structure level 2: Untouched <*sub-step*> element.
- Status element: After clicking the link in G, the status on the right side will change to *done*. When a <*sub-step*> element is clicked, all related Action Primitives are loaded and stay active as long as they are not completed or the <*sub-step*> element is closed manually. When the user achieves all constraints, the element will change to *done*. These elements can also get the status *open* (not clicked or solved yet) or *blocked* (when some predefined constraints are not met).
- Status element: number of uncompleted sub-steps.

## Architecture and implementation

In this part, we describe the architecture and implementation of TVB-EduPack and its integration into TVB. EduPack seamlessly integrates into the existing TVB architecture and GUI by operating on an intermediate level between the front- and back-end components of the existing framework to ensure highest possible independence of specific back-end implementations. During startup, TVB invokes EduPack by loading an XML file that contains a description of its menu structure and a reference to its design definition. Upon clicking on its link, located in the lower right corner of TVB, EduPack becomes active and visible as an overlay that resumes with the last saved configuration and progress.

### Basic architecture of TVB-EduPack and integration into TVB

**Figure 5:**
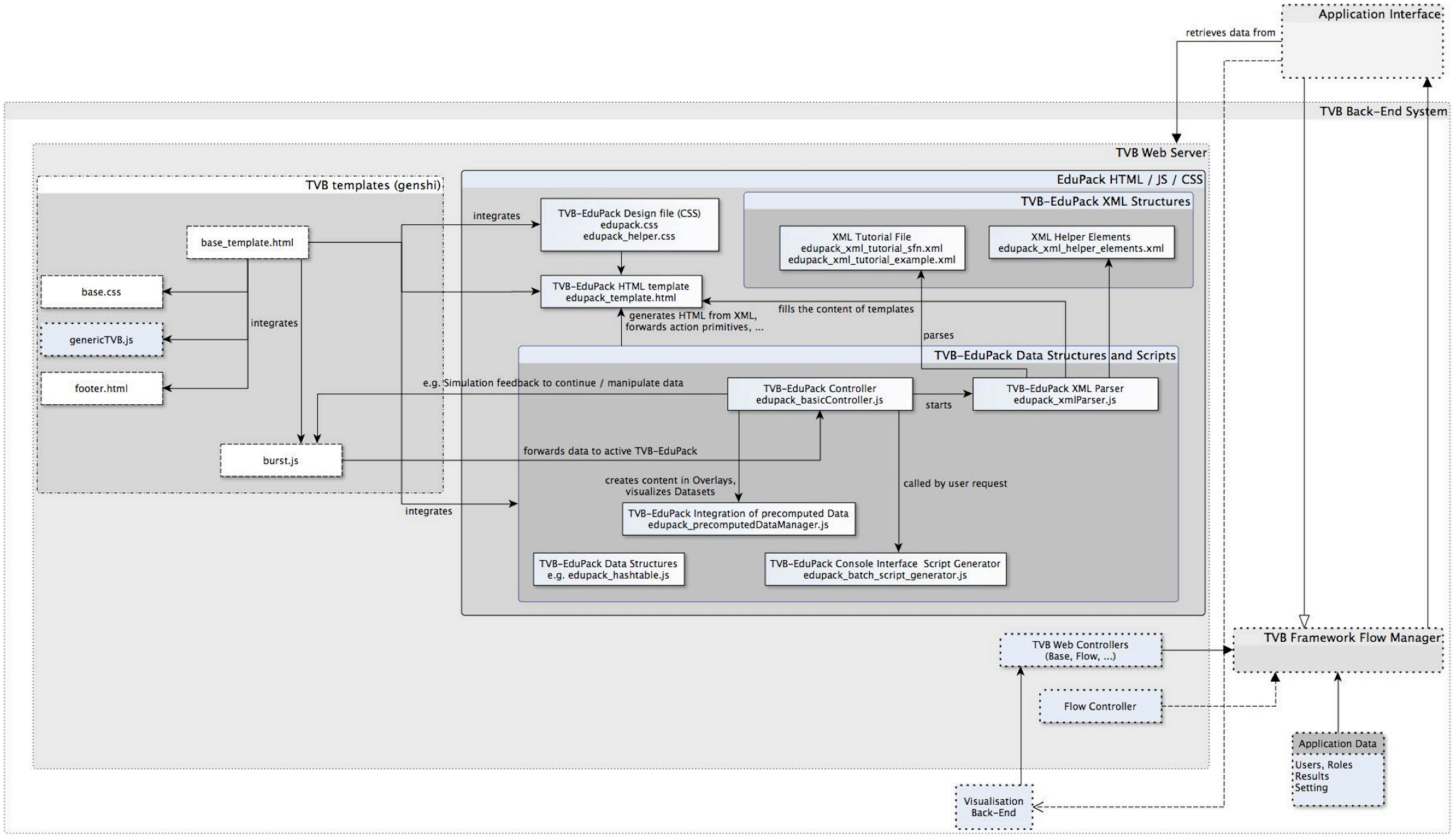
**Excerpt of TVB-framework component diagram with newly integrated EduPack modules and modified TVB modules**.

TVB-EduPack is implemented by a collection of interacting JavaScript scripts, XML Schema Definitions, a HTML template and a CSS design definition file. Figure 5 shows an excerpt of the TVB back-end system that contains the modules of TVB relevant for EduPack, the added EduPack modules and modules of TVB that have been modified for integrating EduPack. As mentioned earlier, the basic components of TVB consist of a graphical HTML application interface (and a command based application interface that implements more functionality than the GUI, but is omitted here since EduPack is solely implemented for the GUI) that is hosted by a web-server in the back-end system. The web server itself is controlled by the so-called Flow Manager that regulates the interaction of all other TVB components (e.g., Simulator component, Upload component, Analyzer component) of TVB.

EduPack is integrated into TVB by adapting relevant HTML and JS templates of TVB's Web Server module. Figure 5 shows native, added and adapted modules: boxes with dotted lines show untouched TVB elements, boxes with solid lines represent files and packages of TVB-EduPack and dashed lines show modified TVB packages. TVB-EduPack modules are only activated by certain triggers in the TVB workflow, e.g., during startup, upon user input, when simulations are started and when so-called Action Primitives verify the chosen parameter configuration. The TVB-EduPack controller module is activated by interrupting TVB control flow in the method *launchNewBurst()* of the script *burst.js*. Furthermore, a function was added to that script to forward the current parameter configuration to TVB-EduPack Controller (e.g., to check if these parameters match with a configuration required by an EduCase).

EduPack is integrated into the TVB GUI in the *base.css* design file by adding the used design specifications and a connection to the activation link in the footer menu specified in the *footer.html* template. The script *edupack.js* and the design file *edupack.css* implement the main EduPack functionalities and were integrated into *base_template.html*, which is the central TVB HTML template that contains the main elements and structure of all generated TVB pages. By integrating EduPack scripts into the basic template, the XML-based EduPack menu structure is invoked during TVB startup. XML files are interpreted by the parsing script *edupack_xml_parser.js*. When TVB-EduPack is activated for the first time, several key-value pairs are created in the *localStorage* object of the browser using the methods *localStorage.getItem(key), setItem(key,value)*, and *removeItem(key)* within the EduPack Controller *edupack_basicController.js*. Thereby, information about the currently opened and finished tasks are saved and updated during utilization of TVB-EduPack, such as the combination of the current tutorial, step and sub-step id. This data is needed and used so that users can continue at the point where they last worked with TVB and TVB-EduPack. Furthermore, the values about current progress (key=latestStep, value=stepId) and completed tasks (key=latestTask, value=taskId) are stored and checked by tasks that require certain preconditions to be fulfilled.

Upon invocation by *burst.js*, the TVB-EduPack controller implemented in the script *edupack_basicController.js* either loads the last used tutorial or the default EduPack startup menu if the *localStorage* Object does not contain the related preference variable of the activated Helper Elements (keys: helper_description, helper_parameter, helper_misc) or the latest progress (keys: latest_step, latest_task) due to the first use of TVB-EduPack. Individual parsers were generated for the different XML Schema Definitions, i.e., Helper Elements, tutorials, task-related preconditions, and Action Primitives. For all Helper Elements and tutorials, the parser creates HTML DOM tree elements and integrates them into TVB-EduPack. The other two parsers are event-driven and check for task-related preconditions and Action Primitives when a task is started. For all found preconditions and Action Primitives the attributes are saved or forwarded to *TVB-EduPack Controller* and TVB workflow continues. When one or more preconditions are not fulfilled, this will be shown by a default, or, if defined, customized message and all other checks are canceled. The controller checks for similar active primitives and either ignores the new primitive, adapts its lifecycle, or adds it to the list of current active Action Primitives. In this way, the EduPack controller manages active primitives and prevents interference between Action Primitives of the same or a different type. Furthermore, *TVB-EduPack Controller* updates active Action Primitives when the user updates form fields or clicks in specific areas. Some of the user's actions can trigger *TVB-EduPack Controller* to activate, review or remove active instances or even to interfere with TVB workflow if required. At the moment parsers do not automatically check for errors in the imported XML files. Therefore, tutorial authors have to ensure that the used elements and their structure are valid and that proper XML well-formedness is given (this can be checked with free tools like http://xmlgrid.net). The script *edupack_precomputedDataManager.js* contains methods to import pre-computed simulation results and to forward parameter settings to the Simulator interface of TVB. The file *edupack_batch_generator.js* implements methods that automatically generate Python scripts for the command line interface of TVB on the basis of simulations that have been configured within the TVB GUI.

### Interactive guides and tutorials

Interactive Guides and Tutorials are implemented using two different types of XML Schema Definitions (XSD): the first one provides a generic Schema for the two types of tutorials (TVB-EduStart and TVB-EduCase), while the other one specifies so-called Helper Elements. Both XSDs are independent from one another and can be created or adapted to aim at different user groups. In contrast to the main EduPack menu, the following elements are only loaded when the user activates those elements.

#### Helper elements

**Figure 6:**
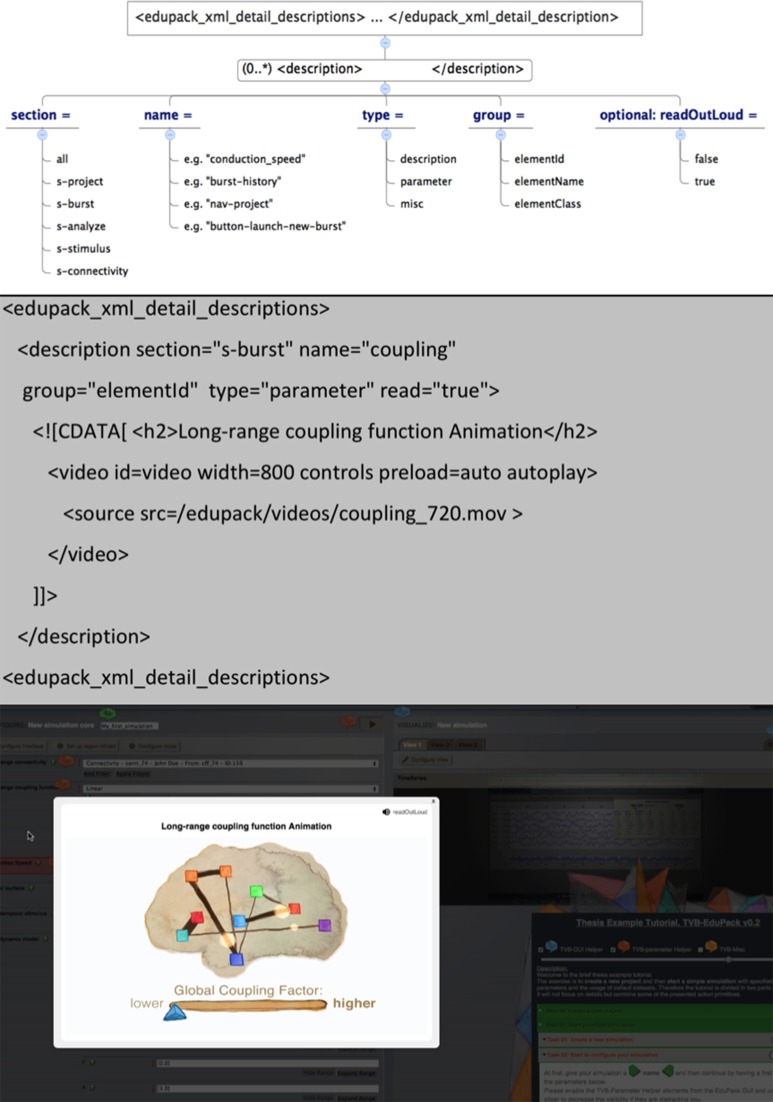
**Helper element XSD, exemplary code listing and resulting Helper element**.

Helper Elements are dialogue balloons that appear in the GUI to give the user three types of information about GUI, modeling parameters and miscellaneous TVB features. In addition to the context sensitive Helper Elements implemented in TVB (green buttons with question marks that show information about data types from the TVB-API), Helper Elements provide in-depth information in a didactic form. The powerful simulation framework offered by TVB comes at the expense of a complex and, especially in the beginning, potentially overwhelming interface. When the user clicks on a TVB-EduPack Helper element, the main screen of TVB gets darkened and a speech bubble appears that reveals the contextual information (Figures 4, 6). Didactic information or instructions come in the form of short video or audio sequences, graphics or text. The size of the speech bubble overlay relates to the content and is opened directly next to the object. The overlay is movable, if it obscures other object in the TVB GUI. HTML tags can be used to emphasize text, integrate images, diagrams or link to videos stored locally or on the web. The Helper element closes when the user clicks on the “*x*” in the upper right corner or into the shaded background. Behavior and appearance is specified in the CSS class edupack.css and JavaScript scripts edupack_xml_parser.js for the integration of the XML Schema described elements and edupack_basicController.js for basic functionality and management.

The XSD top-level element for Helper Elements is the <*edupack_xml_detail_description*> *tag*. Helper element text is defined as content of the <description> tag, while specification of Helper element type and parameters is done by setting respective attributes of that tag as summarized in Figure 6. It is recommended to use the *![CDATA[…content…]]*-tag for text in order to integrate HTML tags, use media elements or special characters. Figure 6 shows the components of the Helper element XSD as well as an example listing and result in the GUI.

In order to create TVB-EduPack Helper Elements, the TVB internal DOM-Tree ID of the elements must be specified by the *name*-attribute. IDs are identified by a browser tool like Firebug or Apache JMeter by hovering over the respective items with the mouse. GUI elements of TVB either have a unique ID in the DOM-Tree or belong to a certain class that can be used to identify the corresponding element. Most of the element IDs or class names are already specified in the Helper Elements XML file (commented out) and can be directly used. This XML document is also a good starting point to create specialized Helper Elements for different user groups, like beginners or specific content for computational neuroscientists (at the moment TVB-EduPack integrates only one type of Helper Elements). Positioning of Helper Elements is done through the retrieved position of the DOM-Tree elements. Resizing or scrolling events in the browser are fetched by the *edupack_basicController.js*, because these might require the repositioning of all visualized elements on screen.

#### EduStart and EduCase tutorials

**Figure 7:**
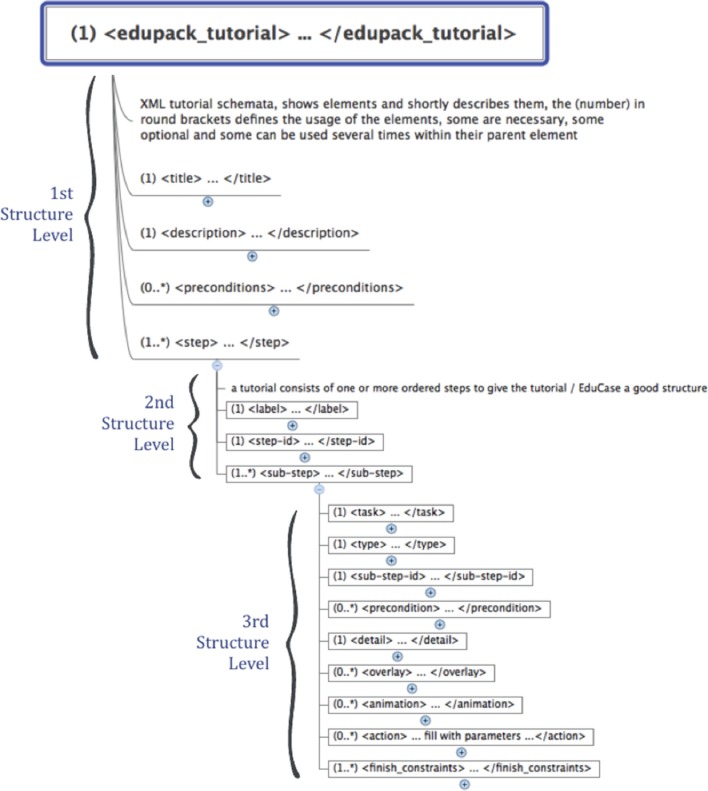
**Overview of the XML Tutorial structure of all three levels**.

Interactive assistance and tutorials allow the user to quickly start working with TVB and to set up own simulations. EduStart and EduCase Tutorials have the advantage that the user is not bound to follow a single strict sequence of steps, but is enabled to leave the suggested pathway of a tutorial and to flexibly continue a started tutorial on an independent path. Tutorials are implemented by a simple XSD and can be quickly created and modified. Figure 7 summarizes the structure of tutorials.

Tutorials are organized into three structural levels comprising mandatory as well as optional elements. All mandatory elements are marked with a leading (1) before their tag-name. As mentioned earlier, at the current stage parsing scripts do not verify the validity of XML files, which might lead to errors and even crashes of TVB. The first structural level is the top level and organizes the individual steps of the tutorial specified by the <step> element. Furthermore, it provides tags for the configuration of general tutorial properties: tutorial title (<*title*> *element*), a description of tutorial content (<*descriptions*> *element*) and preconditions for tutorials (<*preconditions*> *element*). The <precondition> element can be used to organize individual tutorials into a collection of tutorials that are sorted by increasing levels of difficulty (tutorials can only be accessed if their preconditions are met). A precondition is set by referencing the content of the <*title*> *element* of another tutorial. The second level is organized as a sequence of one or more <*step*> *tags*, which correspond to the sequence of tutorial steps. Mandatory elements are the <*label*> *tag* and the <*step-id*> *tag*, which specify the heading of the step that will be shown in the menu respectively the number according to which it will be arranged and for referencing sub-level elements and animations within the menu. The top element of the third level is the <*sub-step*> *tag*. Each second-level <*step*> *element* capsules at least one <*sub-step*> *element*. Mandatory elements are: a <*sub-step-id*> *tag* sorted by increasing numbers, a <*task*> *tag* that contains the title of the step and a <*type*> *tag* that specifies the color of the sub-step (i.e., “green” for description, “red” for parameter or “yellow” for miscellaneous) and all related links, overlays or pointers.

**Figure 8:**
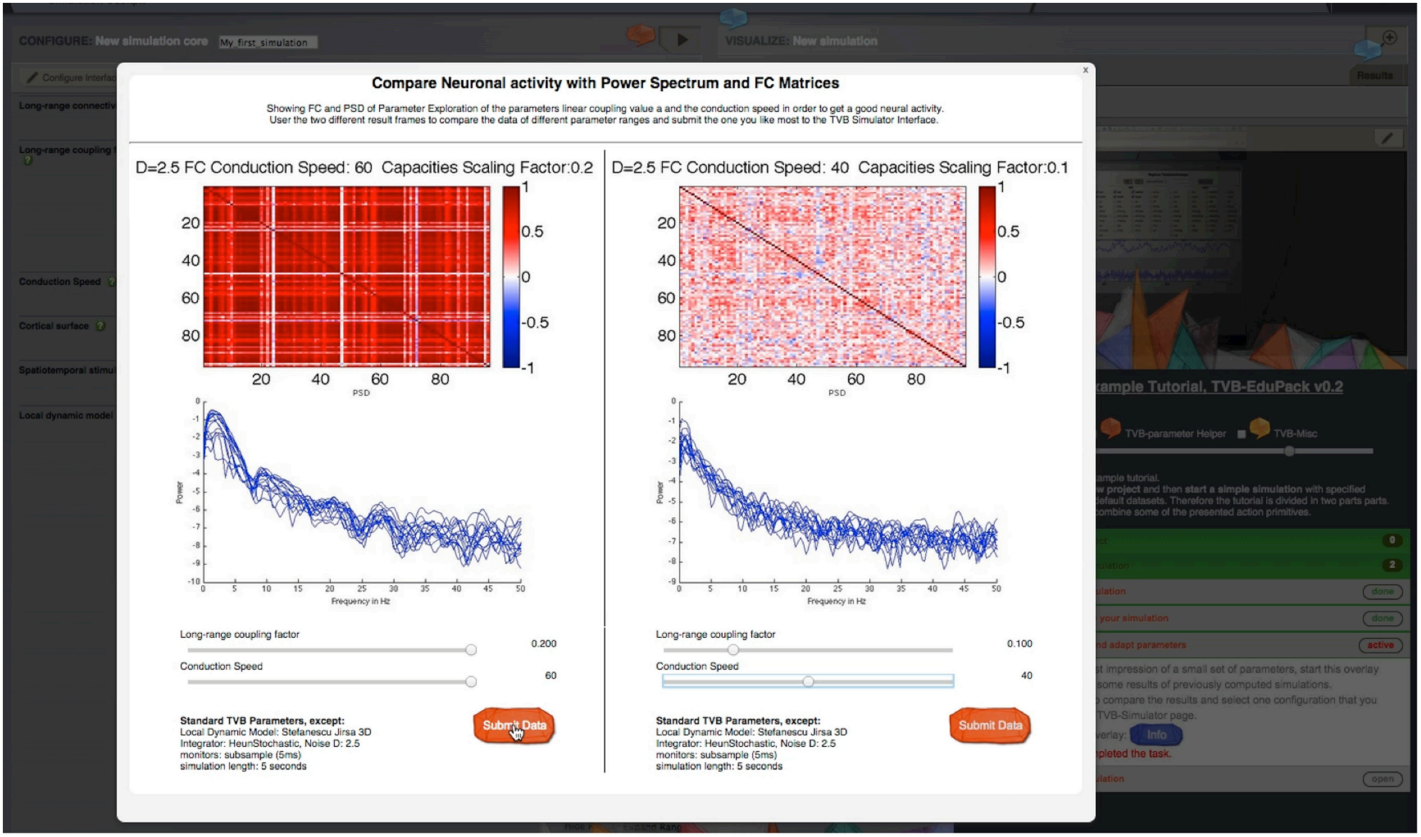
**Parameter regime exploration tool: Sliders are used to set different simulation and model parameters**. Pre-computed simulation results show the associated dynamics for different metrics of interest like FC or power spectral density. Thereby, users can quickly determine meaningful parameter ranges for their own simulations.

A <*sub-step*> element becomes visible when the user activates its parent step element. Upon clicking on <*sub-step*>, the element becomes active and reveals its content, if all <*preconditions*> of that <*sub-step*> element are fulfilled. Preconditions are used when the execution of the current task depends on the successful completion of a previous task, e.g., when the user is asked to switch to another interface in TVB. If a <*precondition*> is not fulfilled, the <*sub-step*> element is marked as “blocked” and will not open. For this case, the author can specify an error message that pops up in a small overlay. The content of the <*precondition*> *tag* defines the error; it can be simple text or images if *CDATA* tags are used. Within <*sub-step*> elements graphical overlays can be specified with the <*overlay*> *tag*. Overlays are a way to incorporate further audio-visual material like videos or animations that describe the scientific or structural backgrounds of a certain step. Figure 8 shows an example of an overlay object with sliders. An overlay appears in the sub-step content area as a blue “*info”*-button that opens the overlay upon clicking. Upon clicking the overlay appears and TVB GUI is darkened. By clicking in the darkened background the overlay closes again. The implementation of overlays is described in detail later.

Another child node of the <*sub-step*> element is the tag <*finish_constraints*>, which is used to set task-related constraints like, e.g., waiting for a key input or validating the parameter configuration by some presets. Constraints are specified by the contents of the <state> tag. In the current version, parameter checks are the only available finish constraint; thus, the only allowed content of the <state> element is *parameter_constraint*. The content of the <manual> tag specifies the text of a link that appears below the task allowing users to manually set the state of the task to “*completed*” and to continue with the next task.

#### Action primitives

**Figure 9:**
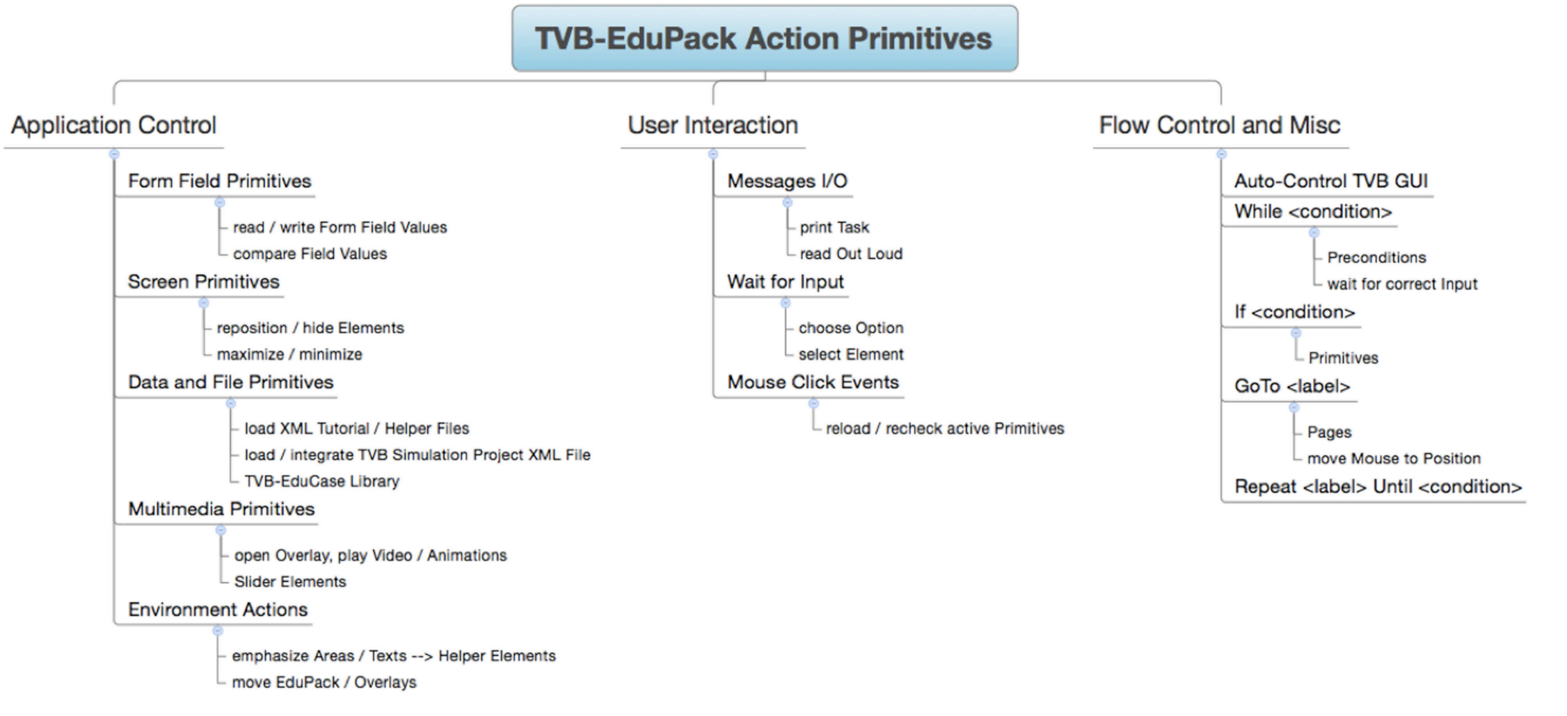
**Several applications of Action Primitives**.

Action Primitives are a set of XML Schema Definitions that specify event-related GUI operations for TVB-EduPack. Applications are, e.g., highlighting of TVB GUI elements, moving of the EduPack to different position within TVB GUI or pointing at elements to hint users at important content. Action Primitives can be flexibly created, modified or combined to allow easy contribution and adjustment by the community. They are integrated in EduPack by a JavaScript script that allows to add or delete Action Primitives or to change their status. During runtime, they are controlled by the so-called action-manager, which schedules incoming or deletes expired Action Primitives. Integration happens at specific levels in the different XML structures of EduPack. When the respective level becomes activated, the action primitive is loaded and started and stays active until certain constraints have been fulfilled or the enclosing element is closed. An overview of Action Primitive's applications is given in Figure 9.

The <*task_overlay*> primitive can be used to point to a selected object on the screen and is mainly used in tutorials, e.g., to hint the user at the location of a previously described functionality. One or more <*task_overlay*> elements are specified within one <*sub-step*> element. Upon activation, TVB-EduPack adds a type-colored TVB-Helper element and a task id number to the selected position. By specifying more than one <*task-overlay*>, it is possible to implement a sequence of such visual pointer elements, e.g., to show a series of GUI actions for carrying out a certain operation. Since <*task_overlay*> can be used in the same manner as <*overlay*>, it is also possible to include animations or videos and to position them at the relevant GUI location, e.g., to illustrate the effect of a certain parameter an explanatory animation can be placed right next to the respective configuration field. Besides the previously used element type for color coding (for this structure called <*helper_type*>), additional parameters are specified as attributes of the <*action*> *element* and can have the same values as the <*action*> *element* used by Helper Elements (Figure 6).

As the name suggests, the <*move_edupack*> primitive allows tutorial authors to move TVB-EduPack to another position, which is useful when the TVB-EduPack menu is hiding relevant elements. In contrast to other Action Primitives that can be integrated multiple times into one <*sub-step*> level, the <*move_edupack*> will only work once in a <*sub-step*> element. After the task is completed, TVB-EduPack will automatically move back to its original position unless the next task includes a <*move_edupack*> primitive as well. This action element is most likely used in combination with other Action Primitives, because its movement allows the user to click elements behind TVB-EduPack without closing it. To implement a horizontal translation operation, the attribute *moveX* is set. If this parameter is not set or defined, a default value of 200px movement to the left will be used for the translation.

The Action Primitive <*highlight_and_compare*> is used to emphasize elements that have values that need to be re-evaluated (e.g., parameter settings that will produce meaningless results) or that need special attention during a tutorial by drawing a red rectangle around them. The element stays highlighted until the number in the related field is changed to the value specified by the *value*-attribute. The action primitive <*highlight*> works similarly, but only highlights elements without checking for a preset. The action primitive <*set_param_value*> overwrites the value of any parameter field within the Simulator interface. The action primitive <*load_project_xml*> loads a TVB simulation XML, compares the specified values with the current values and highlights all non-matching fields using the action primitive <*highlight_and_compare*>.

In Table 1, we summarize all Action Primitives that have been implemented up to now and allowed attributes and values.

**Table 1:** **Action types and corresponding attributes and values**.

**Action type**	**Attributes**	**Values**
*Task_overlay*	*Section*	e.g., “s-project,” “s-burst,” …
	*Helper_type*	“task”
	*Group*	“elementId” or “elementClass”
	*Variable_name*	“nav-project,” “coupling,” …
*Move_edupack*	*Section*	e.g., “s-project,” “s-burst,”…
	*MoveX (optional)*	Numeric Value; default: 200
*Highlight*	*Section*	e.g., “s-project”
	*Variable_name*	e.g., “coupling”
*Highlight_and_compare*	*Section*	e.g., “s-project”
	*Variable_name*	e.g., “coupling”
	*Value*	Numeric value
*Load_project_xml*	*Section*	“s-burst”
*Set_param_value*	*Group*	“elementId” or “elementClass”
	*Variable_name*	e.g., “coupling,” “simulation_length,” …
	*Value*	Numeric value

### Console interface script generator

The Console Interface Script Generator creates a Python command line script for the console interface of TVB upon button press. Upon setting a parameter configuration with the Simulator Interface, users can download a script that performs the specified simulation. When the user hits the corresponding button in the Script Generator Menu of TVB-EduPack, the method *onButtonCreateBatchPressed()* (which is associated with the button) in the script *edupack_batch_generator.js* is triggered and calls the function *checkAndRunTVBPage()*, which checks whether the user is on the Simulator page of TVB. If not, an overlay appears that informs the user that he is not on the right page. If the Simulator interface is active, the method *runBatchGeneratorSimulator()* is activated. This method reads out the parameters from the simulator page using the function *getSubmittableData()* from the TVB script *genericTVB.js* (functions are accessible by their linkage in *base_template.html* cf. Figure 5). Afterwards, the function saves all parameter variable names and value pairs into a hash table data structure. After that, the function *createScript()* fills the parameter keys and values into a command script template. Finally, the method *saveTextAsFile()* saves the script as text file and triggers an automatic download of the file within the browser.

## Conclusions and future work

The TVB neuroinformatics platform provides versatile possibilities for construction, simulation and analysis of subject-specific brain models and can be operated by a graphical and a command interface. Here, we introduced an expandable educational module for the neuroinformatics platform TVB dubbed TVB-EduPack. In order to enable users to quickly learn GUI operations, EduPack gathers interactive user guides, tutorials, and other educational content (see also Supplementary Figures 1, 2). Step-by-step tutorials guide novice and experienced users through basic TVB operation and complex use-case scenarios. Different types of interactive elements are implemented that can be used for a variety of functions, e.g., event listeners that check parameter settings for validity as they are entered, graphical highlighting items lead users' attention to relevant parts of the GUI or point out further options. Multimedia content like animations, videos, audio, images and text can be integrated at each stage of the TVB workflow. In order to ease the transition from the GUI to the more powerful and flexible programming interface, EduPack automatically creates command scripts on the basis of simulations that were configured within the GUI.

EduPack's architecture was designed with the intention of creating a lightweight, generic and extensible framework that enables convenient ongoing content development. The main interface used for the communication with TVB-EduPack is implemented at the HTML level of TVB, reducing invasion of its core libraries to a minimum. On this layer, GUI objects are organized in a DOM tree, allowing simple addressing and manipulation. The core functionality of EduPack is implemented by JavaScript scripts that are loaded during the startup of TVB and that register event handlers to TVB's HTML objects to catch desired events and to interrupt the flow of TVB. EduPack's content is defined by XML structures that allow the quick and easy authoring of tutorials and other features for the development of entirely new functionalities. Therefore, EduPack represents a convenient interface to add new GUI extensions and functionality to TVB at its top layer without requiring manipulation of TVB's source code.

To initially estimate the impact of EduPack, several tests regarding hardware consumption and usefulness were performed. Activation of EduPack did not produce recognizable differences in the rendering performance of TVB's GUI. Runtime and CPU consumption tests did neither for the web browser process, nor for the TVB process show noticeable differences. A comparison of initialization speed at the startup of TVB and execution speed of simulations showed no difference for an instance that was patched with EduPack and one that was not. Additional main memory usage with activated EduPack is below one Megabyte. EduCases that contain animations or audio-visual content are varying in size (up to several MB) depending on their contents, but are only loaded for the time of execution of the respective EduCase. Hard disk consumption of EduPack (including animations) is at the present stage around 350 MB.

The impact of EduPack was tested with EduStart for six subjects (five medical students and one computer science student) that had no prior experience with TVB and that were only provided with minimal instructions about the purpose of the software and how to activate TVB and EduPack. It took the students on average 35 min to complete EduStart, upon which they were able to use basic TVB functionalities. EduStart covers tasks like creating a new project, uploading subject data, performing a simulation (containing an explanation of the mathematical model, the biophysical interpretation of parameters and the integration scheme, details on how to select a structural connectome, parameters from the simulator interface, setting of output monitors like BOLD or EEG and starting the simulation) and performing initial analysis of the results (i.e., viewing simulated time series).

Besides the EduStart tutorial, EduCases are available that provide step-by-step guidance for using advanced TVB functionalities like opening and viewing of simulation results, usage of different viewing options, exporting of results, generating, and analyzing different types of neuroimaging signals and the design and application of electrical stimulation paradigms. A further EduCase explains how structural connectomes are integrated in the model, how they are used within the software and how they can be modified to emulate certain conditions like lesions of brain regions. Other EduCases cover compound scenarios that employ simultaneous application of different functionalities like monitoring and analysing the results of stimulation or lesion studies with different modalities. The TVB team is developing new EduCases in an ongoing manner and we encourage the community to participate in this process by providing own EduCases for the emerging TVB applications.

Although the chosen XML-based approach supports generality and flexibility, it could represent an obstacle for XML-naive users. Therefore, to make authoring for EduPack more convenient, upcoming versions will integrate a graphical tool that automatically generates XML files by directly recording GUI interaction, as well as drag-and-drop tools to integrate EduPack elements like overlays, sliders or action elements. Another upcoming feature, dubbed “Autopilot,” allows users to watch demos of TVB use cases and workflows in the GUI.

## Obtaining TVB-EduPack

TVB-EduPack is made available as part of the main TVB distribution, which can be obtained from the TVB website www.thevirtualbrain.org. To get in touch with other users and developers and for questions concerning usage and development the TVB mailing list can be reached at https://groups.google.com/forum/#!forum/tvb-users. For contributing own code, the GitHub code repository can be found at https://github.com/the-virtual-brain. Online documentation and programming interface specification is located at http://docs.thevirtualbrain.org.

## Author contributions

HM, MS, DV, SR, AL, RR, PT, LD, JM, AS, VJ, AM, and PR conceived the toolbox, developed the toolbox, provided content for the toolbox, and wrote the manuscript.

### Conflict of interest statement

The authors declare that the research was conducted in the absence of any commercial or financial relationships that could be construed as a potential conflict of interest.

## Supplementary material

Supplementary Figure 1 **Description of TVB-EduPack menu: After a user finished all tasks of a section, the number should be zero**. Here, the user opened the next section—it indicates four open tasks while one of them is already active. All elements are colored red/orange as indicator for the parameter-type related tasks.

Supplementary Figure 2 **The tutorial shows the user how to create a new simulation and offers to read the text out loud**.

## References

[B1] Becker R. Knock S. A. Ritter P. Jirsa V. (2015). Relating alpha power and phase to population firing and hemodynamic activity using a thalamo-cortical neural mass model. PLoS Comput. Biol. 11:e1004352. 10.1371/journal.pcbi.1004352 26335064 PMC4559309

[B2] Cabral J. Hugues E. Sporns O. Deco G. (2011). Role of local network oscillations in resting-state functional connectivity. Neuroimage 57, 130–139. 10.1016/j.neuroimage.2011.04.010 21511044

[B3] Deco G. Jirsa V. McIntosh A. Sporns O. Kötter R. (2009). Key role of coupling, delay, and noise in resting brain fluctuations. Proc. Natl. Acad. Sci. U.S.A. 106, 10302–10307. 10.1073/pnas.0901831106 19497858 PMC2690605

[B4] FitzHugh R. (1961). Impulses and physiological states in theoretical models of nerve membrane. Biophys. J. 1, 445–466. 10.1016/S0006-3495(61)86902-6 19431309 PMC1366333

[B5] Jansen B. H. Rit V. G. (1995). Electroencephalogram and visual evoked potential generation in a mathematical model of coupled cortical columns. Biol. Cybern. 73, 357–366. 10.1007/BF00199471 7578475

[B6] Jirsa V. K. (2009). Neural field dynamics with local and global connectivity and time delay. Philos. Trans. R. Soc. A 367, 1131–1143. 10.1098/rsta.2008.0260 19218155

[B7] Jirsa V. Sporns O. Breakspear M. Deco G. McIntosh A. R. (2010). Towards The Virtual Brain: network modeling of the intact and the damaged brain. Arch. Ital. Biol. 148, 189–205. 21175008

[B8] Kuramoto Y. (1975). Lecture Notes in Physics, International Symposium on Mathematical Problems in Theoretical Physics. New York, NY: Springer-Verlag.

[B9] Matzke H. (2014). TVB-EduPack—An Interactive Learning and Scripting Platform for the Virtual Brain (Master's thesis). Free University of Berlin. 10.3389/fninf.2015.00027 PMC4658631 26635597

[B10] Ritter P. Schirner M. McIntosh A. R. Jirsa V. K. (2013). The Virtual Brain integrates computational modeling and multimodal neuroimaging. Brain Connect. 3, 121–145. 10.1089/brain.2012.0120 23442172 PMC3696923

[B11] Roy D. Sigala R. Breakspear M. McIntosh A. R. Jirsa V. K. Deco G.. (2014). Using The Virtual Brain to reveal the role of oscillations and plasticity in shaping brain's dynamical landscape. Brain Connect. 4, 791–811. 10.1089/brain.2014.0252 25131838

[B12] Sanz-Leon P. Knock S. A. Spiegler A. Jirsa V. K. (2015). Mathematical framework for large-scale brain network modelling in The Virtual Brain. Neuroimage 111, 385–430. 10.1016/j.neuroimage.2015.01.002 25592995

[B13] Sanz-Leon P. Knock S. A. Woodman M. M. Domide L. Mersmann J. McIntosh A. R.. (2013). The Virtual Brain: a simulator of primate brain network dynamics. Front. Neuroinform. 7:10. 10.3389/fninf.2013.00010 23781198 PMC3678125

[B14] Schirner M. Rothmeier S. Jirsa V. K. McIntosh A. R. Ritter P. (2015). An automated pipeline for constructing personalized virtual brains from multimodal neuroimaging data. Neuroimage 117, 343–357. 10.1016/j.neuroimage.2015.03.055 25837600

[B15] Spiegler A. Jirsa V. (2013). Systematic approximations of neural fields through networks of neural masses in The Virtual Brain. Neuroimage 83, 704–725. 10.1016/j.neuroimage.2013.06.018 23774395

[B16] Stefanescu R. A. Jirsa V. K. (2008). A low dimensional description of globally coupled heterogeneous neural networks of excitatory and inhibitory neurons. PLoS Comput. Biol. 4:e1000219. 10.1371/journal.pcbi.1000219 19008942 PMC2574034

[B17] Wilson H. R. Cowan J. D. (1972). Excitatory and inhibitory interactions in localized populations of model neurons. Biophys. J. 12, 1–24. 10.1016/S0006-3495(72)86068-5 4332108 PMC1484078

[B18] Wong K.-F. Wang X.-J. (2006). A recurrent network mechanism of time integration in perceptual decisions. J. Neurosci. 26, 1314–1328. 10.1523/JNEUROSCI.3733-05.2006 16436619 PMC6674568

[B19] Woodman M. M. Pezard L. Domide L. Knock S. A. Sanz-Leon P. Mersmann J.. (2014). Integrating neuroinformatics tools in TheVirtualBrain. Front. Neuroinform. 8:36. 10.3389/fninf.2014.00036 24795617 PMC4001068

