# Supplementary figures and images for: TVB-EduPack—An Interactive Learning and Scripting Platform for The Virtual Brain
Source: Front Neuroinform. 2015 Nov 25;9:27. doi: 10.3389/fninf.2015.00027 (PMC4658631; doi:10.3389/fninf.2015.00027)

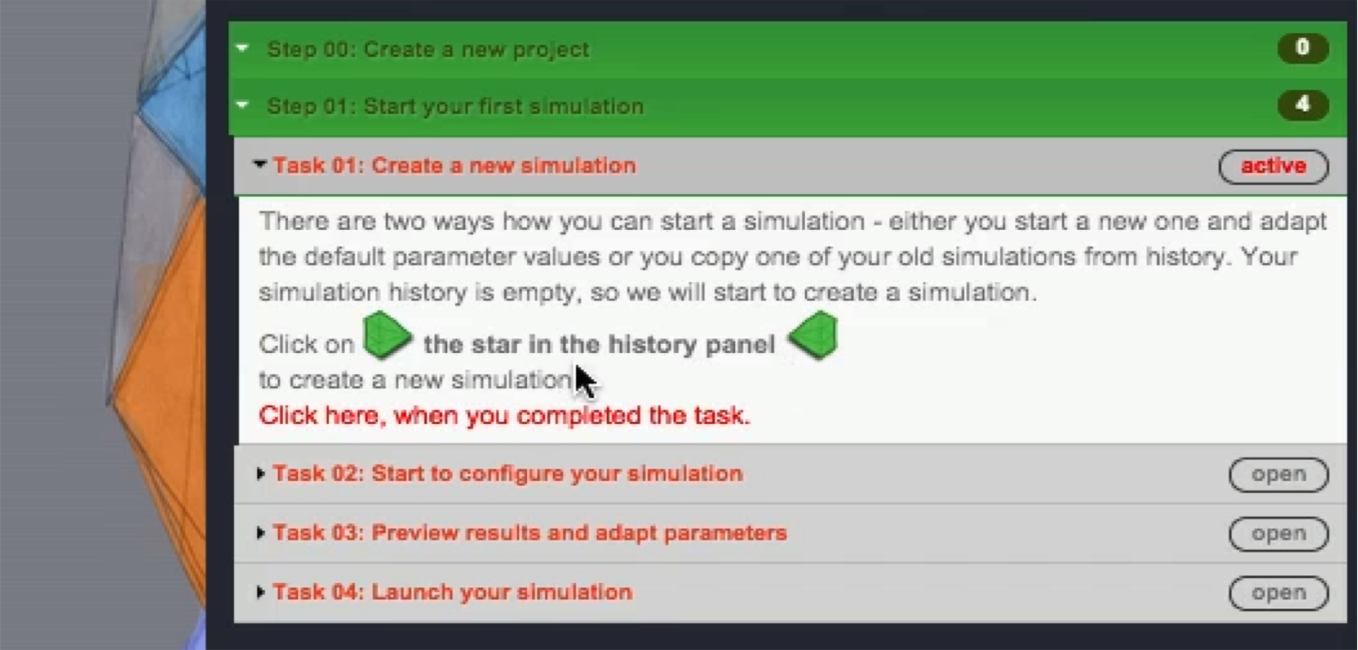

Supplement: Supplementary Figure 1 — Description of TVB-EduPack menu: After a user finished all tasks of a section, the number should be zero. Here, the user opened the next section—it indicates four open tasks while one of them is already active. All elements are colored red/orange as indicator for the parameter-type related tasks. [file Image1.TIFF]

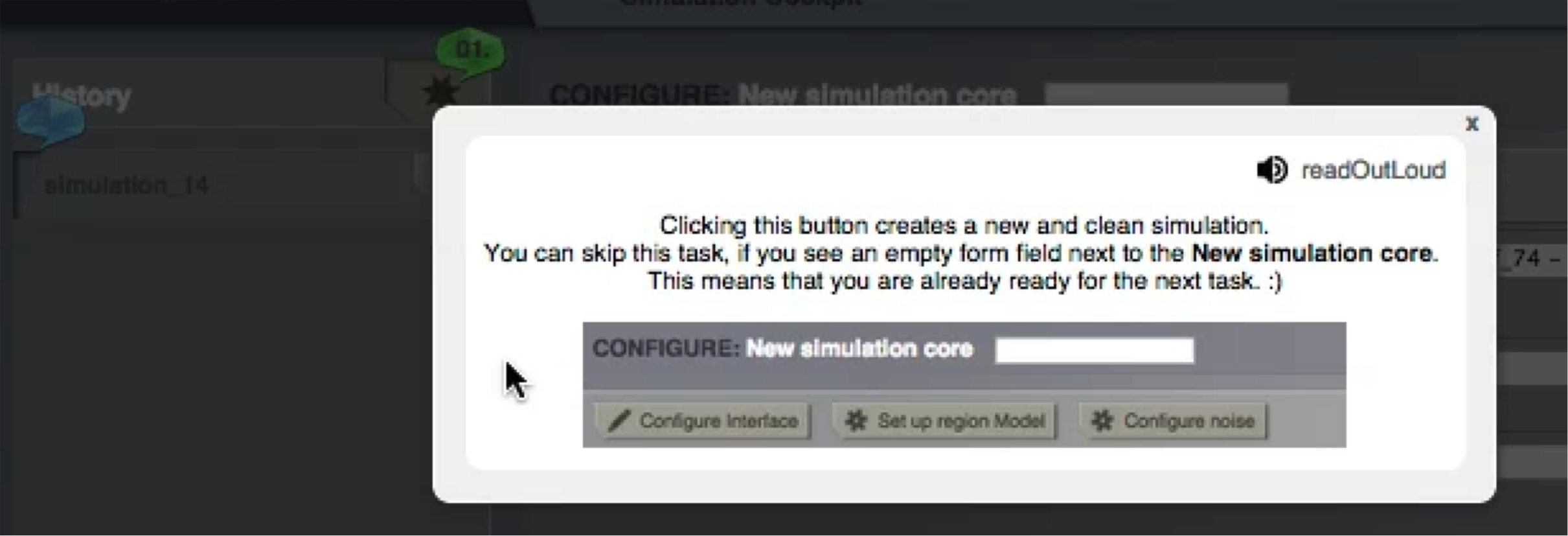

Supplement: Supplementary Figure 2 — The tutorial shows the user how to create a new simulation and offers to read the text out loud. [file Image2.TIFF]
